# Transcriptomic profiling of urine extracellular vesicles reveals alterations of CDH3 in prostate cancer

**DOI:** 10.18632/oncotarget.6899

**Published:** 2016-01-12

**Authors:** Felix Royo, Patricia Zuñiga-Garcia, Verónica Torrano, Ana Loizaga, Pilar Sanchez-Mosquera, Aitziber Ugalde-Olano, Esperanza González, Ana R. Cortazar, Laura Palomo, Sonia Fernández-Ruiz, Isabel Lacasa-Viscasillas, Maria Berdasco, James D. Sutherland, Rosa Barrio, Amaia Zabala-Letona, Natalia Martín-Martín, Amaia Arruabarrena-Aristorena, Lorea Valcarcel-Jimenez, Alfredo Caro-Maldonado, Jorge Gonzalez-Tampan, Guido Cachi-Fuentes, Manel Esteller, Ana M. Aransay, Miguel Unda, Juan M. Falcón-Pérez, Arkaitz Carracedo

**Affiliations:** ^1^ CIC bioGUNE, Bizkaia Technology Park, Biscay, Spain; ^2^ Centro de Investigación Biomédica en Red de Enfermedades Hepáticas y Digestivas (Ciberehd), Spain; ^3^ Department of Urology, Basurto University Hospital, Bilbao, Spain; ^4^ Department of Pathology, Basurto University Hospital, Bilbao, Spain; ^5^ Cancer Epigenetics and Biology Program, Bellvitge Biomedical Research Institute (IDIBELL), Barcelona, Spain; ^6^ Ikerbasque, Basque Foundation for Science, Bizkaia, Spain; ^7^ Biochemistry and Molecular Biology Department, University of the Basque Country (UPV/EHU), Bizkaia, Spain

**Keywords:** extracellular vesicles, exosomes, prostate cancer, urine biomakers

## Abstract

Extracellular vesicles (EV) are emerging structures with promising properties for intercellular communication. In addition, the characterization of EV in biofluids is an attractive source of non-invasive diagnostic, prognostic and predictive biomarkers. Here we show that urinary EV (uEV) from prostate cancer (PCa) patients exhibit genuine and differential physical and biological properties compared to benign prostate hyperplasia (BPH). Importantly, transcriptomics characterization of uEVs led us to define the decreased abundance of Cadherin 3, type 1 (CDH3) transcript in uEV from PCa patients. Tissue and cell line analysis strongly suggested that the status of CDH3 in uEVs is a distal reflection of changes in the expression of this cadherin in the prostate tumor. CDH3 was negatively regulated at the genomic, transcriptional, and epigenetic level in PCa. Our results reveal that uEVs could represent a non-invasive tool to inform about the molecular alterations in PCa.

## INTRODUCTION

In the recent years, the search of biomarkers in urine has focused on the characterization of urinary extracellular vesicles (uEVs), trying to overcome the complexity and variation of this biofluid [1, 2]. Under the denomination of uEVs, there is a complex mixture of vesicles, including exosomes, microvesicles and apoptotic bodies [3, 4]. Although there are no clear markers to distinguish them, exosomes are defined as small membrane vesicles with a diameter of 40–150 nm formed by inward budding of the membrane of late endosomes resulting in the formation of multivesicular bodies (MVB) fulfilled of intraluminal vesicles. Then, some of these (MVB) fuse to the plasma membrane releasing in this manner the exosomes to the extracellular *milleu* [5]. Microvesicles or ectosomes refer to plasma membrane shedding vesicles of 0.1–1 μm [6]. Apoptotic bodies are assumed to be of bigger size [7]. uEVs are released by several tissues along the urinary tract and their cargo varies depending on their origin [8]. Evidence of the presence of uEVs belonging to prostate has been already reported [9, 10] and the cargo includes proteins of prostate origin such as prostate-specific membrane antigen (PSMA) [11]. Proteomic analysis of uEVs in PCa patients has been recently carried out with promising results as a source of biomarkers [12] and the use of microRNAs as markers for this disease have been also extensively reported and reviewed [13]. Most of the studies to date focus on the comparative analysis of healthy and PCa patients. This raises the question of the existence of biomarkers that can discriminate PCa from BPH [14], a pathology that has been shown to interfere with well established biomarkers such as prostate-specific antigen (PSA) [15]. In the present work, we aimed at identifying PCa biomarkers within uEVs through the analysis of the uEV transcriptome. We selected transcripts with a presence-absence pattern in BPH and PCa, and we extensively validated the candidate transcript encoded by the *Cadherin 3, type 1* gene (CDH3). Importantly, we corroborated this observation in a miniaturized assay that could facilitate the translation of the results into the clinic. Finally, the analysis of mRNA in prostate tumor tissue from patients revealed alterations in this gene, coherent with genomic transcriptional and epigenetic changes, all pointing at the inhibition of CDH3 in PCa. Overall, our results support that analysis of uEVs could represent a non-invasive method to evaluate and monitor PCa alterations.

## RESULTS

### Characterization of uEVs from BPH and PCa patients

**Figure 1:**
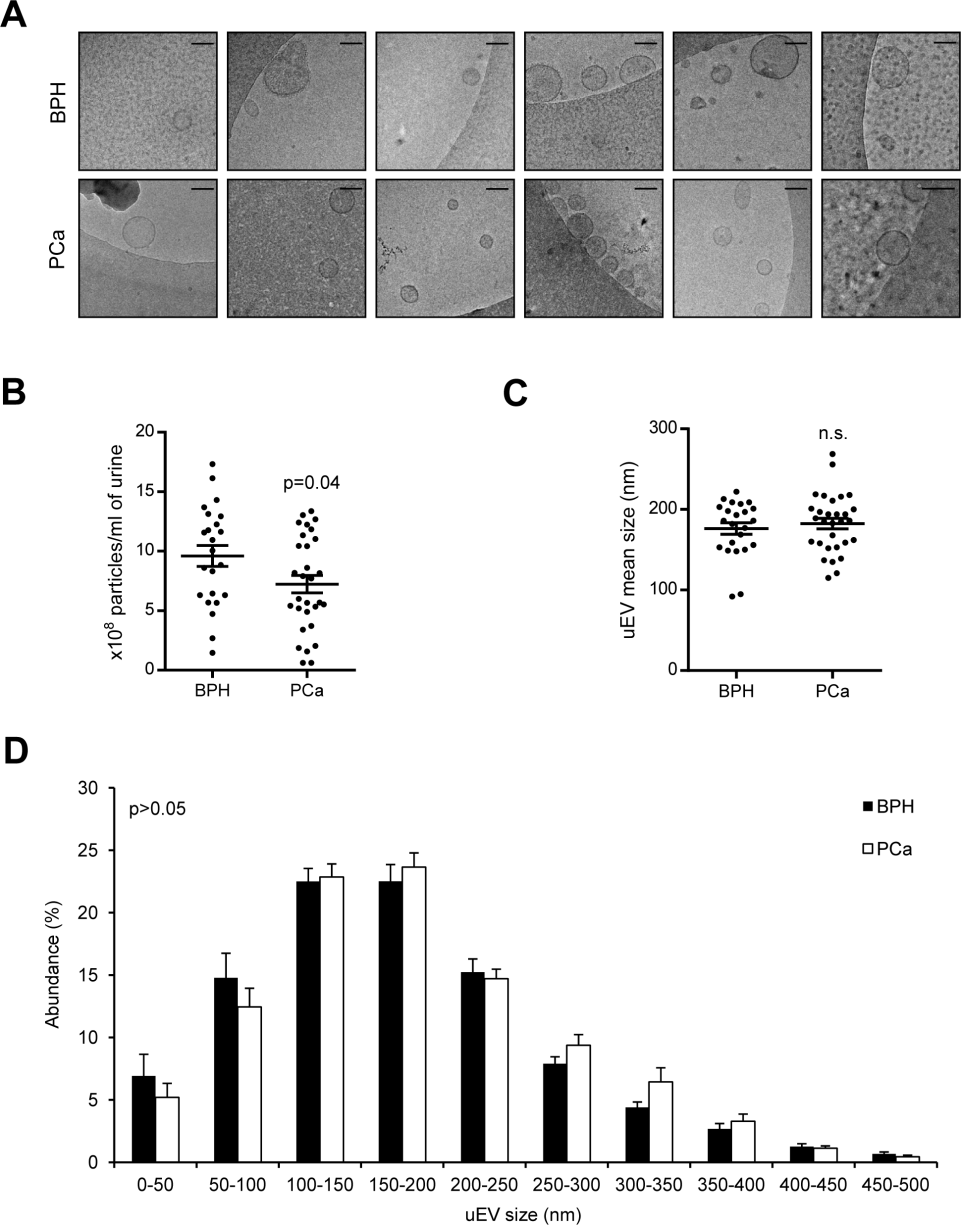
Physical characterization of uEVs from PCa and BPH samples (**A**) Representatives cryo-TEM micrographs of uEVs isolated from BPH and PCa urine samples. *Bar*, 100 nm. *n* = 3. (**B** and **C**) Box-plots showing number (B) or size (C) of particles isolated from each group, indicating the mean and s.e.m. (*n* = 23 fo BPH and 30 for PCa). (**D**) Size distribution of the particles isolated from each preparation (Mean ± s.e.m. is depicted, *n* = 23 fo BPH and 30 for PCa). Statistic test: Student *t* test.

As a first approach, we analyzed the physical characteristics of uEVs from patients with BPH and PCa by comparing more than 23–30 independent preparations from each group (Supplementry Table S1). In order to validate the ultracentrifugation procedure [16] for isolation of uEVs, the presence of double membrane vesicles by cryo-electron microscopy (Figure 1A) and EV markers by western blot [28] was confirmed (Supplementary Figure S1). We next analyzed uEV size and number in urine of BPH and PCa patients. Nanoparticle-tracking analysis (NTA) was performed in samples before and after urine ultracentrifugation. NTA-estimated particle number was comparable before (8.9e^10^ ± 1.47e^10^ particles/ml in BPH, and 9.3e^10^ ± 1.29e^10^ particles/ml in PCa; mean ± s.e.m.; *n* = 5; *p* > 0.05) and was reduced in PCa after ultracentrifugation (2.49e^8^ ± 2.46e^7^ particles/ml in BPH, and 1.56e^8^ ± 1.69e^7^ particles/ml in PCa; mean ± s.e.m.; *p* = 0.04) (Figure 1B). However, no significant changes were observed in particle size before (217 ± 13.2 nm in BPH, and 215.8 ± 6.9 nm in PCa; mean ± s.e.m.; *n* = 5; *p* > 0.05) or after ultracentrifugation (176.6 ± 6.7 nm in BPH, and 182.4 ± 6.9 nm in PCa; mean ± s.e.m.; *n* = 5; *p* > 0.05) (Figure 1C). It is worth noting that NTA analysis in samples before ultracentrifugation could detect non-uEV particles and contaminants as positive events (and hence explain the larger number and average size) while after filtration and ultracentrifugation the values obtained are more representative of an uEV-enriched preparation. Although no statistically significant differences were found, NTA analysis revealed a trend to a different size distribution of the uEVs, with a lower abundance of small vesicles (0–100 nm) and a greater abundance of large (150–200 nm) and very large (250–350 nm) vesicles in PCa when compared with BPH (Figure 1D). Of note, we observed a size discrepancy between TEM and NTA analysis of uEVs. Although it warrants further investigation, this fact is probably due to two main factors: the technology employed by NTA to determine particle size and the potential effect of the TEM sample preparation protocol on this parameter.

Further to this characterization, we analyzed the changes in cargo in BPH and PCa. RNA concentration per vesicle was comparable in BPH and PCa uEVs (0.017 ± 0.006 ng RNA per million uEVs in BPH and 0.0046 ± 0.0005 ng RNA per million uEVs in PCa; mean ± s.e.m.; *n* = 9–10; Mann Whitney U *p* = 0.13). Similarly, we did not observe significant differences in protein concentration (0.041 ± 0.01 μg protein per million uEVs in BPH and 0.019 ± 0.003 μg protein per million uEVs in PCa; mean ± s.e.m.; *n* = 9–10; Mann Whitney U *p* = 0.18).

### Transcriptomic analysis of PCa and BPH uEVs

**Figure 2:**
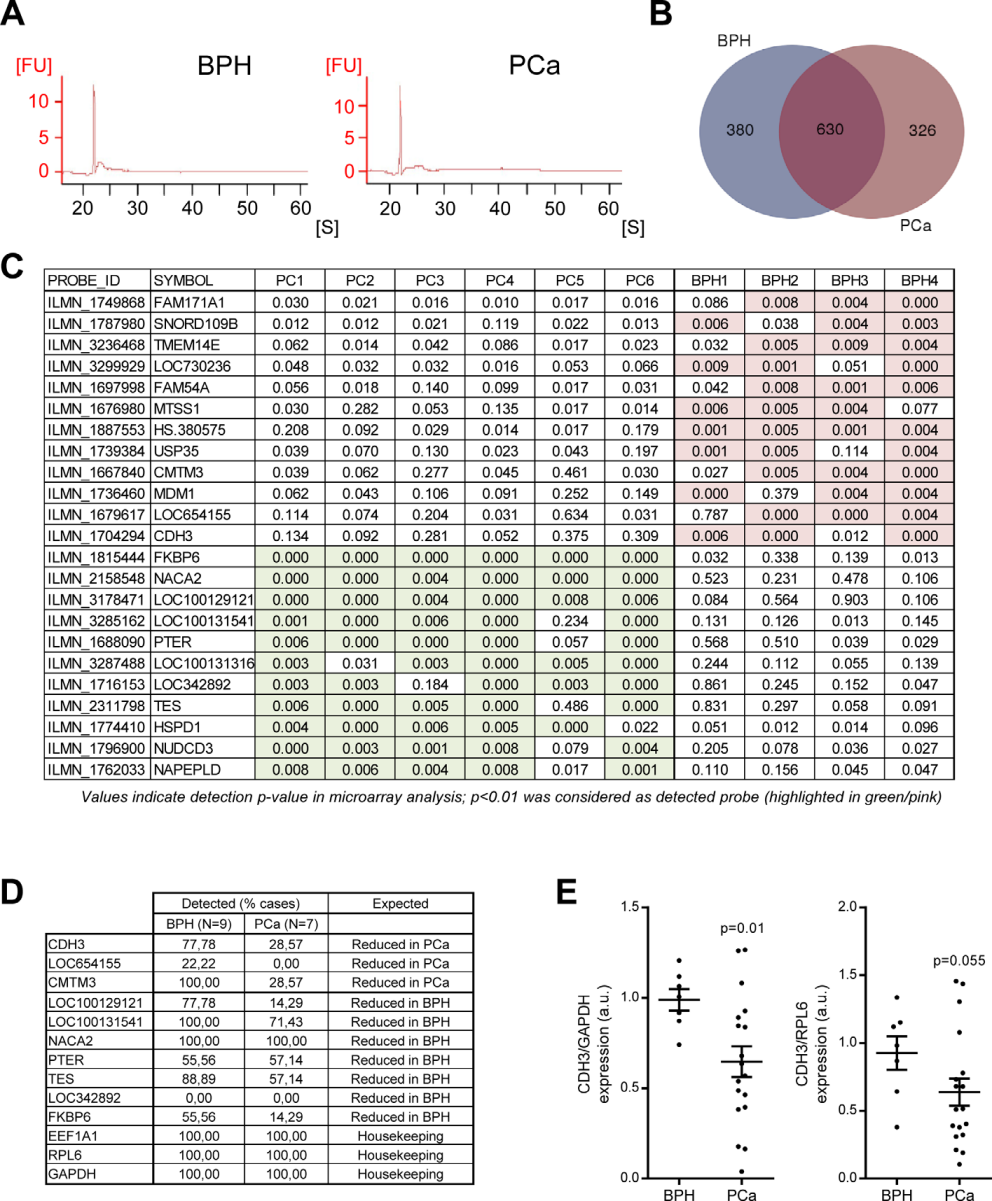
Transcriptomic analysis of uEVs reveals transcripts with differential abundance in BPH and PCa (**A**) Representative analysis of RNA size distribution obtained from the Bioanalyzer analysis of uEV preparations. *n* = 4–6. (**B**) Venn diagram depicting the number of unique transcripts identified in each experimental condition (*n* = 4 for BPH and *n* = 6 for PCa). (**C**) Transcripts exhibiting a presence-absence pattern in BPH and PCa. The transcripts shown complied with the requirements of being absent in one condition and with a minimum presence of 75% of cases in the other. Detection *p*-value is presented, where a limit was established in *p* < 0.01 in the microarray analysis (significant conditions are highlighted in pink or green in BPH and PCa, respectively). (**D**) Detection of ultracentrifugation-purified candidate uEV transcripts by qRTPCR. Detection was established as consistent amplification in the technical settings employed in the assay. *n* = 7 for BPH and *n* = 9 for PCa. (**E**) Transcript abundance of CDH3 relative to GADPH (*left*) and RPL6 (*right*) in Norgen-purified uEVs-associated RNA samples. *n* = 6–7 for BPH and *n* = 18 for PCa. FU: fluorescence units. S: seconds. Error bar represents s.e.m. a.u. = Arbitrary units. Statistic test: Mann Whitney *U* test (E).

We next aimed at identifying molecular alterations in uEV cargo from PCa patients. It has been recently reported that these particles present a genuinely differential proteome in patients harboring PCa [12]. However, little is known about the transcript content of uEVs and the potential of these molecules to inform about the biological characteristics of PCa, especially when comparing to patients with BPH. To address this question, we extracted RNA of uEVs from BPH and PCa patient samples. First, we observed lack of overt changes in overall RNA size distribution (Figure 2A). Next, we labeled and hybridized BPH and PCa uEV-derived RNA into whole genome Illumina gene expression microarrays. The results showed the detection (detection *p*-value < 0.01) of 1336 unique transcripts in the two groups analyzed (presence in 50% of the cases in either group was defined as positive, Supplementry Table S3), 1010 in BPH and 956 in PCa (Figure 2B). Venn analysis revealed an overlap of 47.1% from total unique transcripts in BPH and PCa (Figure 2B). We performed a further step in candidate transcript selection by identifying genes that were selectively detected in one of the two biological settings (BPH or PCa, in at least 75% of the cases). Illumina platform provides information about the probability of a probe to present a signal that is different to background noise, for which purpose we established a confidence interval of 99% (*p* < 0.01). The list of differentially detected probes is shown in Figure 2C. In addition, we took advantage of the microarray analysis in order to define housekeeping genes that would have similar abundance in uEVs from BPH and PCa patients. To this end, starting from normalized signal values, we defined genes with no differential abundance (*p*-value > 0.95 and fold change no greater than ± 5%; Supplementry Table S4). From this analysis, we selected two transcripts, Eukaryotic Elongation Factor 1A1 (EEF1A1) and Ribosomal Protein L6 (RPL6), that we monitored in subsequent studies. In addition, we also included Glyceraldehyde Phosphate Dehydrogenase (GAPDH) as a housekeeping gene supported by prior studies of our group [16].

### Validation of uEV biomarkers of PCa

To ascertain the potential of candidate uEV transcripts, we performed qRTPCR from an independent set of ultracentrifuge-purified uEV retrotranscribed RNA (using an average of 1.5e^7^ uEVs per reaction). Firstly, the abundance of housekeeping transcripts (RPL6, EEF1A1, GAPDH) was strongly correlated (Supplementry Figure S2A), reinforcing the notion of their value as housekeeping transcripts. The use of these controls allowed us to identify 4 cases with lack of amplification in all three transcripts, which was considered an exclusion criterion for the analysis. Secondly, the evaluation of 10 transcripts of interest (From Figure 2C) revealed that two candidates, Cadherin 3, type 1 (CDH3) and CKLF-Like MARVEL Transmembrane Domain Containing 3 (CMTM3), exhibited the predicted behavior in the validation dataset (Figure 2D). These two transcripts were predominantly detected in BPH uEVs, whereas the detection rate was below 30% in PCa uEVs. Of note, we confirmed that these transcripts were contained in uEVs, since they exhibited resistance to RNase treatment (Supplementary Figure S2B).

Our results demonstrate that we can identify transcripts with differential abundance in PCa uEVs, employing 50 mL of urine and using an ultracentrifugation-based method for uEV isolation [16]. However, biomarker identification requires miniaturization of the assay with the consequent scaling down of the starting material. To refine our detection method, we employed a commercial exosomal RNA purification procedure (Norgen Biotek) in an independent set of samples that allowed us to reduce urine volume to 10 mL. We then performed qRTPCR from Norgen-purified retrotranscribed RNA. We evaluated the expression level of the two best candidates, CMTM3 and CDH3. As shown before, the two housekeeping transcripts employed (GAPDH and RPL6) exhibited a strong and significant correlation (Supplementry Figure S2C). Interestingly, this purification method precluded detection of CMTM3, while recapitulated the reduction in CDH3 with higher sensitivity using normalization against RPL6 (0.69 ± 0.1; mean ± s.e.m.; *p* = 0.055) and GAPDH (0.65 ± 0.08; mean ± s.e.m.; *p* = 0.01) (Figure 2E).

Taken together, our transcriptomic analysis reveals that CDH3 abundance is reduced in PCa uEVs and sets the basis for PCa biomarker search based on uEV transcript analysis.

### uEVs are indicators of PCa alterations

Our results convincingly show that CDH3 abundance is reduced in uEVs from PCa patients. On the basis of these results, we hypothesized that the alteration observed in uEVs might be a reflection of transcriptomic changes in the prostate tumor.

**Figure 3:**
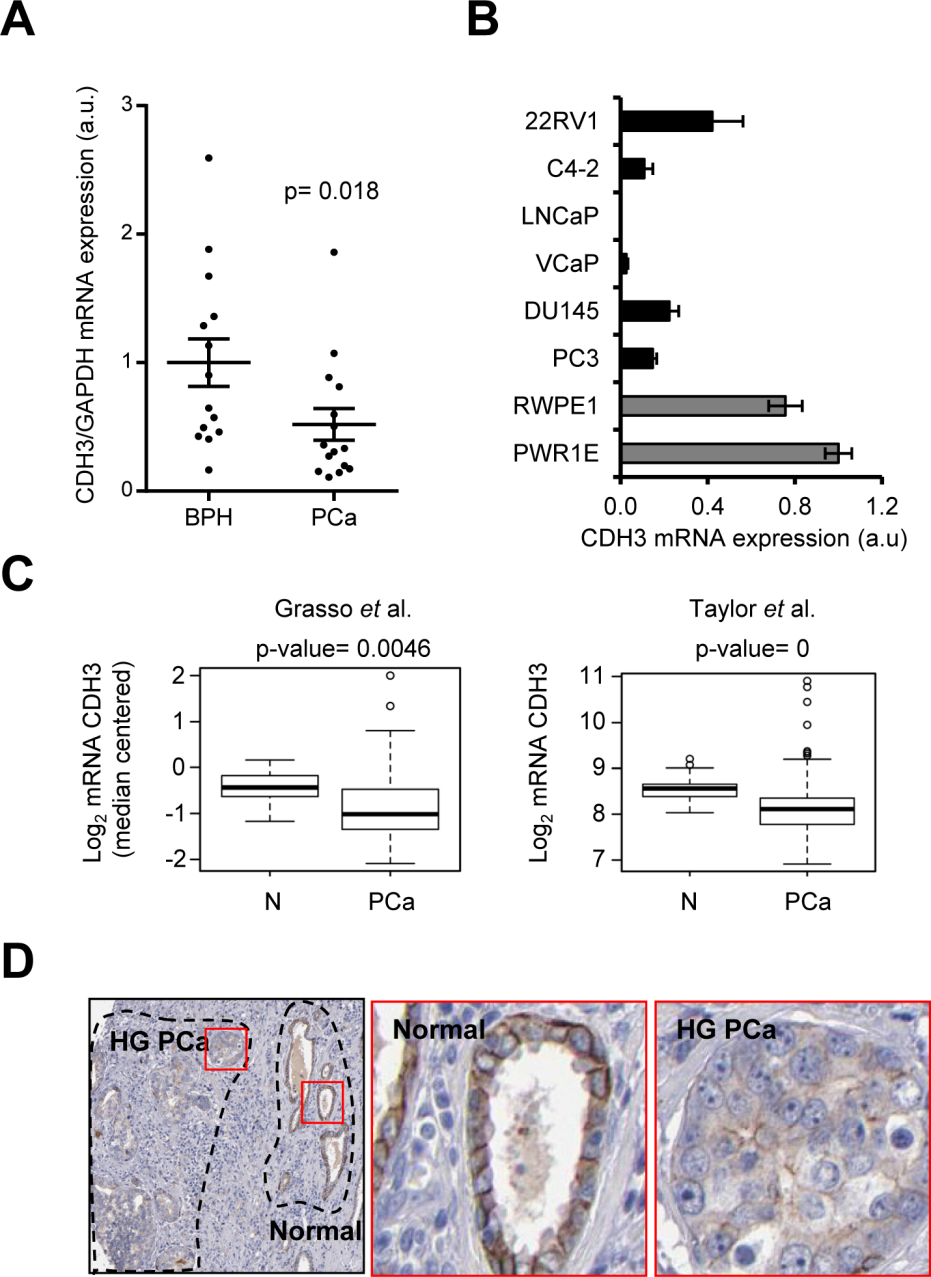
CDH3 expression is reduced in PCa specimens (**A**) CDH3 expression in tissue biopsies from BPH and PCa. CDH3 expression relative to GAPDH is shown. *n* = 14 for BPH and *n* = 15 for PCa. (**B**) CDH3 expression in a panel of metastatic prostate cancer cell lines (black bars) and benign immortalized prostate cell lines (grey bars) relative to beta-Actin. *n* = 3. (**C**) CDH3 expression in two PCa datasets (Taylor PCa *n* = 150, normal *n* = 29; Grasso PCa *n* = 76, normal *n* = 12). (**D**) Representative images of immunohistochemical detection of CDH3 protein in PCa. Middle panel corresponds to a normal area and right panel to high grade PCa (HG PCa). Data source: Human Protein Atlas. Statistic test: Mann Whitney *U* test (A), Student *t* test (C).

In order to confirm our hypothesis, we studied the expression of CDH3, in a set of BPH and PCa tissue specimens. The results of CDH3 expression analysis demonstrated that it was significantly decreased in tissue from patients with PCa compared to BPH (0.52 ± 0.12; mean ± s.e.m.; *p* = 0.018), in full coherence with our observation in uEVs (Figure 3A). Of note, these results could lead to the notion that the association between transcriptomic tumor cell landscape and exosome RNA cargo correlate at high frequency. However, prior studies from our lab showed that known cancer genes, such as PTEN, do not exhibit a direct correlation between uEVs mRNA abundance and PTEN tumor alterations [16], suggesting a selective process in cargo loading into uEVs.

Next, we ascertained the potential extrapolation of this observation to other biological contexts, such as a panel of benign prostate cells and metastatic prostate cancer cell lines and large human PCa datasets. Interestingly, the expression of CDH3 in prostate cell lines revealed a down-regulation of the transcript in metastatic cancer cell lines (black), compared to benign-immortalized cells (grey) (0.17 ± 0.07; mean ± s.e.m.) (Figure 3B). Importantly, this observation was confirmed in two datasets where the expression of PCa specimens was compared to biopsies from healthy patients [24, 25] (Figure 3C) and was in full agreement with a previous report [29].

We also monitored the expression levels of other transcripts identified in uEVs. On the one hand, CMTM3 expression, which was shown to be down-regulated in the ultracentrifugation uEVs (but not detected with Norgen extraction method), showed a significant reduction in PCa compared with BPH tissues, but this result was not reproduced in publicly available PCa datasets and exhibited only a modest trend in PCa cell lines (Supplementary Figure S3A–S3C). On the other hand, our housekeeping genes RPL6 and EEF1A1 showed no consistent alterations throughout the same analytical layout (Supplementary Figure S3A–S3C).

We next ask whether the reduction of CDH3 expression observed in PCa could be extrapolated to other urogenital cancers. Data mining analysis was performed in bladder and renal cancer datasets (www.oncomine.org, [30]). Although there was certain consistency in the alteration of CDH3 expression within the same tumor type, the directionality of the alterations was not preserved among the different tumor types (Supplementary Figure S3D).

In order to address whether gene expression alterations in CDH3 could be translated in a decrease in the protein expression, we took advantage on publicly available initiatives for immunoreactivity analysis. Proteinatlas (www.proteinatlas.org, [31–35]) allows the visualization of immunohistochemistry (IHC) staining in a wide array of tissues. There was data available for CDH3 staining with high quality IHC-specific antibodies. Importantly, the staining in normal prostate epithelia corroborated the staining of basal prostate epithelial cells, in agreement with reports in this and other epithelial tissues [29, 36, 37] (Figure 3E, middle panel and Supplementary Figure S3F). As predicted, CDH3 expression was decreased in PCa specimens. This result was particularly evident in tumor samples with adjacent non-neoplastic tissue (Figure 3D). Interestingly, CDH3 sub-cellular distribution was altered in tumor cells, with a predominant loss of membrane immunoreactivity (Figure 3D).

**Figure 4:**
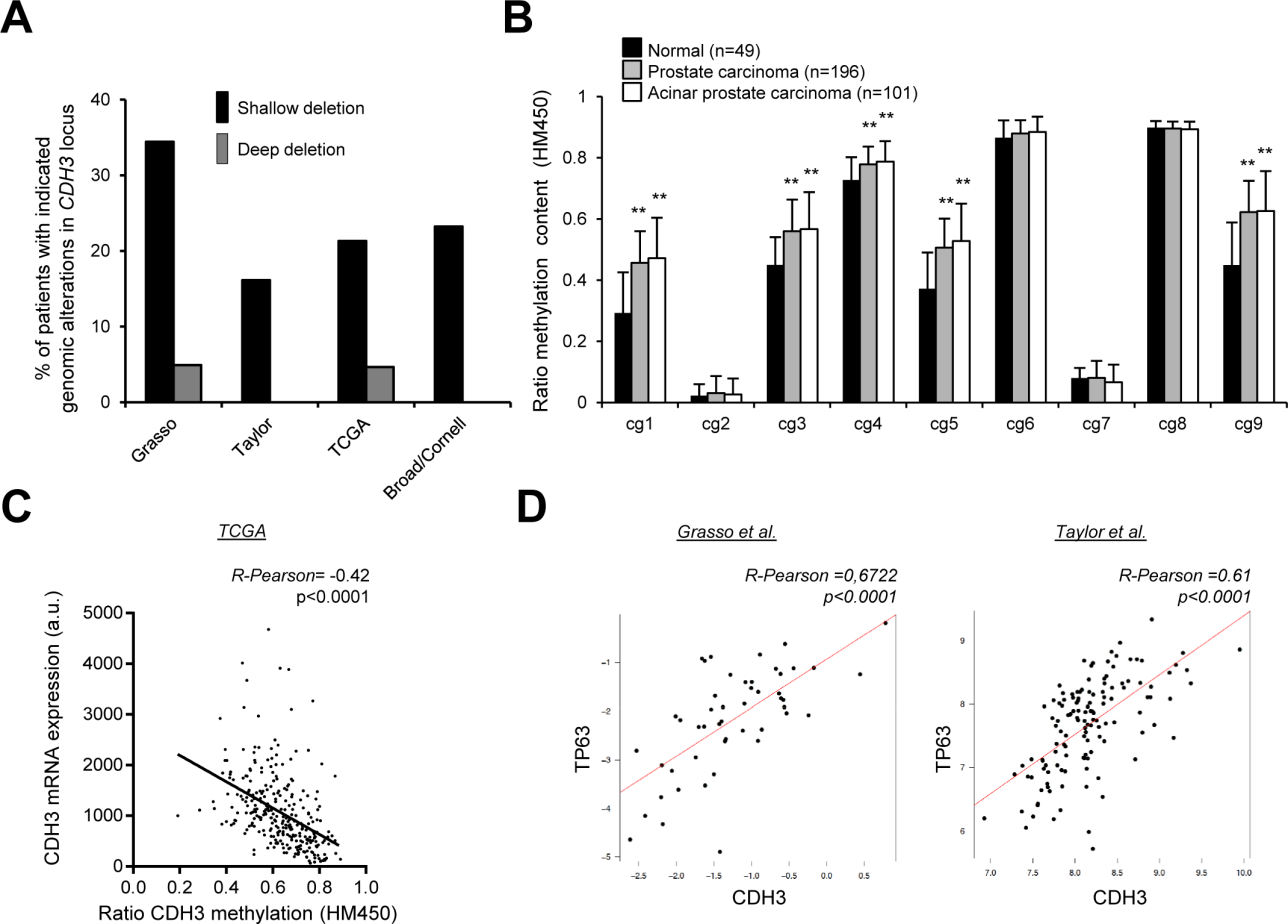
Evaluation of the molecular events accounting for CDH3 down-regulation in PCa (**A**) Analysis of the genomic alterations in *CDH3* locus in four PCa databases (Taylor *n* = 93, Grasso *n* = 61, TCGA *n* = 258, Broad *n* = 56). (**B** and **C**) Promoter methylation analysis from TCGA database evaluating methylation in *CDH3* locus (B) *n* = 49 for normal tissue, *n* = 101 acinar PCa and *n* = 196 for PCa) and the correlation between methylation status and CDH3 mRNA expression (**C**) *n* = 294). (**D**) Correlation analysis between CDH3 and Tp63 expression in two independent datasets. (Grasso, *n* = 49; Taylor, *n* = 131; primary tumors). Statistic test: Student *t* Test (B); Pearson's coefficient (*R*) (C, D).

We next asked the molecular cues leading to the down-regulation of CDH3 in PCa. On the one hand, we studied the genomic and epigenetic changes occurring in *CDH3* locus. The genomic analysis showed frequent shallow deletions of *CDH3* in four independent PCa datasets (Figure 4A, [24, 25, 38, 39]). Moreover, epigenetic analysis of *CDH3* promoter indicated increased methylation in PCa and a correlation between the methylation status of the locus and the transcript abundance (Figure 4B, 4C; [38, 39]), in line with a previous report [29]). On the other hand, we evaluated the association of CDH3 expression with well-known upstream regulators. Tp63 is a basal prostate epithelial marker which is down-regulated in PCa specimens [40–42], and that has been reported to regulate CDH3 expression [43]. We found a strong correlation between the mRNA expression of Tp63 and CDH3 in prostate specimens, which suggests that transcriptional regulation of this cadherin downstream p63 is at play in PCa (Figure 4D). Altogether, our results indicate that genomic loss, transcriptional regulation and promoter methylation contribute to the down-regulation of CDH3 in PCa.

## DISCUSSION

Extracellular vesicles including exosomes have been detected and characterized in urine [2, 44, 45]. These vesicles vary in composition and are associated with different diseases [12, 46]. Importantly, recent evidence suggests that PCa might exhibit alterations in the composition of uEVs [12, 47, 48]. The majority of the studies are carried out comparing healthy individuals with PCa patients. It is worth noting that there is an increasing incidence of BPH in association with age [14], and the interference that this might introduce to biomarker identification is poorly understood. To address this question, we have performed a transcriptomics analysis comparing the mRNA content of uEVs from patients with BPH or PCa. The results reveal that urine from these two groups have significant alterations in vesicle number. Little is known about alterations in EV production in different pathologies as compared to the nature of its cargo and this aspect warrants further investigation. Importantly, we found a markedly different transcriptomics profile in uEVs from BPH and PCa. We were able to reduce a whole genome analysis (which revealed 1336 transcripts detected in uEV preparations) to two candidate transcripts (CMTM3 and CDH3) with decreased abundance in PCa. Interestingly, the miniaturization of the assay employing an alternative purification method revealed that CMTM3 detection is sensitive to the approach used. This suggests that the detection of uEV transcripts might be affected by the uEV purification protocol and calls for further refinement and characterization of the selectivity and specificity of the uEV isolation methods.

Placental cadherin (P-Cad or CDH3) has been widely studied in cancer [37, 49–59]. This protein regulates cell-cell adhesion processes and cellular differentiation. Interestingly, both oncogenic and tumor suppressive activities of this gene have been described in tissue-specific manner [37, 49–59]. We observe that CDH3 mRNA levels are down-regulated in PCa. This is coherent with preliminary observations at the protein level. It has been suggested that CDH3 is down-regulated and exerts tumor suppressive functions in hepatocellular carcinoma [50] and a prior study reported changes of CDH3 in PCa [29]. Our data suggest that CDH3 may be exerting tumor suppressive activities in PCa.

We show that the regulation of CDH3 expression in PCa occurs at multiple levels. On the one hand, genomic and epigenetic analysis strongly suggests that deletion and methylation of the locus accounts for changes in expression. On the other hand, we find potential regulations at the level of upstream transcriptional regulators. Prior studies showed that CDH3 is a basal epithelial cell marker [29, 37]. Interestingly, Tp63, an upstream regulator of CDH3 [36], presents similar behavior to our gene of interest. Tp63 localizes to basal epithelial cells and is down-regulated in PCa [40–42, 60]. Our correlation analysis in public PCa supports the notion that p63 is a transcriptional upstream regulator of CDH3.

Of note, immunoreactivity analysis has provided preliminary evidence of mis-localization of CDH3 in PCa cells compared to non-tumoral counterparts, Interestingly, this alteration is also observed in other cancers and is associated to poor prognosis [59].

Altogether, our data show multiple means of regulation (genomic loss, DNA methylation, transcriptional regulation, and protein mis-localization) that could potentially lead to the alteration of CDH3 function in PCa.

The function of EVs in cell communication and cancer aggressiveness has emerged in the past years [61, 62]. While their use as source of biomarkers is under intense investigation, there is limited evidence about their potential role as readouts of the tumoral genetic alterations [9]. This study informs about the properties of uEVs to reflect genetic alterations in the tumor of origin. We find that the decrease in abundance of CDH3 in uEVs is coherent with mRNA changes in the prostate tumor cells. This data opens new avenues in the non-invasive characterization of genetic alterations in PCa using uEVs, with the consequent potential for patient stratification.

## MATERIALS AND METHODS

### Patient samples and cell lines analysis

All urine samples were obtained from the Basque Biobank for research (BIOEF, http://www.biobancovasco.org, Basurto University Hospital) upon informed consent and with evaluation and approval from the corresponding ethics committee (CEIC code OHEUN11-12 and OHEUN14-14). Inclusion criteria: For BPH patients, samples were obtained from cases with normal PSA, with symptomatic alterations (polyuria, distress), and that were scheduled for surgery. For PCa cases, samples were obtained from patients with primary localized cancer diagnosed *de novo* and that were scheduled for radical prostatectomy. Urine (40–100 ml) was collected by spontaneous urination between 8–10 AM, in fasting conditions. Patient information, tumor characteristics and urine volume is described in Supplementry Table S1. For prostate tissue specimens, samples were prepared and diagnosed as described in [16]. Cell lines were cultured as described in [17] and RNA was harvested in conditions of exponential growth.

### Urine extracellular vesicle purification

uEV isolation by ultracentrifugation was performed as described in [16]. Briefly, urine was centrifuged at 2000 × g for 5 min to remove cell debris and filtered through 0.22 μm pore-filter before frozen at −80°C. For uEV isolation sample was thawed and subjected to two sequential centrifugations of 11500 × g for 30 min and second 118000 × g for 90 minutes. The pellet containing uEVs was resuspended in 150 μl of cold PBS and frozen for later processing. RNase treatment was not performed unless otherwise specified.

### Western blot

Western blot was performed as described [18], using CD26 (Abcam, Cambridge, UK), CD63 (clone H5C6; from Developmental Studies Hybridoma Bank, Iowa, US), CD13 (clone 3D8; from Santa Cruz Biotechnology Inc.), FLT1 (clone 18; from BD Biosciences) and AQP2 (Sigma-Aldrich) antibodies.

### Transmission electron microscopy (TEM) analysis

For cryo-electron microscopy, uEV preparations were directly adsorbed onto glow-discharged holey carbon grids (100 Holey carbon film of Cu with mess 200; Quantifoil^®^, Germany). Grids were blotted at 95% humidity and rapidly plunged into liquid ethane with the aid of VITROBOT (Maastricht Instruments BV, The Netherlands). Vitrified samples were imaged at liquid nitrogen temperature using a JEM-2200FS/CR transmission cryo-electron microscope (JEOL, Japan) equipped with a field emission gun and operated at an acceleration voltage of 200 kV.

### Size analysis and size distribution

Size distribution within uEV preparations was analyzed by nanoparticle-tracking analysis (NTA) by measuring the rate of Brownian motion using a NanoSight LM10 system (Malvern, U.K.), which is equipped with a fast video capture and particle-tracking software. NTA post-acquisition settings were kept constant for all samples, and each video was analyzed to give the mean, mode, and median vesicle size, as well as an estimation of the concentration [19]. For each preparation, two videos of 30 seconds each were taken. For each video, at least 200 tracks were completed in post-capture tracking analysis.

### Transcriptomic analysis

Total RNA isolation from uEV was achieved by RNeasy columns (Qiagen, Inc). The integrity, size and quantification were evaluated in RNA Pico Chips (Bioanalyzer; Agilent Technologies). For transcriptomic analysis of mRNA-associated uEVs, Illumina whole genome (HumanHT-12_V4.0; DirHyb, nt) method was used as reported [20]. cRNA synthesis was obtained out of 2–25 ng of Total RNA, with TargetAmp^™^ Nano-g^™^ Biotin-aRNA Labeling Kit for the Illumina^®^ System (Epicentre, Cat# TAN07924) and subsequent amplification, labelling and hybridization were performed according to “Whole-Genome Gene Expression Direct Hybridization” Illumina Inc.'s protocol, except the hybridization cRNA concentration, which was 285 ng instead of the standard 750 ng. Raw expression data were background-corrected, log2-transformed and quantile-normalized using the lumi R package [21], available through the Bioconductor repository. Probes with a “detection *p*-value” lower than 0.01 in at least one sample were regarded as detected.

### Retrotranscription and quantitative real time PCR analysis

To extract RNA from uEVs isolated by ultracentrifugation, we employed miRCURY™ RNA Isolation Kit Cell & Plant (Exiqon). In average, 1.5e^7^ vesicles were used per retrotranscription reaction. In addition, a set of samples was extracted by Norgen Biotek Exosomal RNA purification kit, following the manufacturers' instructions. For cell lines, RNA was extracted using NucleoSpin^®^ RNA isolation kit from Macherey-Nagel (ref: 740955.240C). cDNA was synthesized from 0.1–1 μg of RNA using Superscript III (Life Technologies) following the manufacturer's recommendations. For prostate tissue samples, RNA was extracted as reported in [16]. Quantitative Real Time PCR (Taqman qRTPCR) was performed as previously described [18]. Universal Probe Library (Roche) primers and probes employed are detailed in Supplementary Table S2. β-ACTIN (Hs99999903_m1) and GAPDH (Hs02758991_g1) housekeeping assays were from Applied Biosystems and showed similar results.

### DNA methylation

Raw intensity *CDH3* DNA methylation was extracted from The Cancer Genome Atlas dataset (https://tcga-data.nci.nih.gov/tcga/) based on Illumina's 450K methylation array. Data analysis from normal tissues (*n* = 49), prostate carcinoma (*n* = 196) and acinar prostate carcinoma (*n* = 101) were included. A three step-based normalization procedure was performed using the lumi [21] package available for Bioconductor [22], under the R statistical environment [23], consisting in color bias adjustment, background level adjustment and quantile normalization across arrays, as specified in [21]. Methylation level (β-value) for each of the 485, 577 CpG sites was calculated as the ratio of methylated signal divided by the sum of methylated and unmethylated signals plus 100. After normalization step, probes related to X and Y chromosomes were removed as well as those containing a SNPs with a frequency > 1% (1000 Genome project) in the probe sequence or interrogated CpG site.

### Bioinformatics analysis and statistics

The following statistical analysis were employed:

Database normalization: all the datasets used for the data mining analysis were downloaded from GEO and subjected to background correction, log_2_ transformation and quartile normalization. In the case of using a pre-processed dataset, this normalization was reviewed and corrected if required.

For *CDH3* genomic analysis, data from PCa patients with copy number alteration information in Taylor [24], Grasso [25], Broad/Cornell [26] and Robinson [27] et al. datasets was extracted from *cbioportal.org*.

### Correlation analysis

Pearson correlation test was applied to analyze the relation between paired genes. From this analysis, Pearson's coefficient (R) indicates the existing linear correlation (dependence) between two variables *X* and *Y*, giving a value between +1 and −1 (both included), where 1 is total positive correlation, 0 is no correlation, and −1 is total negative correlation. The *p*-value indicates the significance of this R coefficient.

### Statistical analysis

Data represent mean ± s.e.m. of pooled experiments unless otherwise stated. For data mining analysis, ANOVA test was used for multi-component comparisons. Student *T* test or Mann Whitney *U* test for two-group parametric or non-parametric comparisons, respectively. The confidence level used for all the statistical analyses was of 0.95 (alpha value = 0.05). Two-tail statistical analysis was applied for experimental design without predicted result and one-tail for validation experiments.

## SUPPLEMENTARY MATERIALS FIGURES AND TABLES







## References

[1] Pisitkun T, Johnstone R, Knepper MA (2006). Discovery of urinary biomarkers. Mol Cell Proteomics.

[2] Pisitkun T, Shen RF, Knepper MA (2004). Identification and proteomic profiling of exosomes in human urine. Proc Natl Acad Sci U S A.

[3] Kalra H, Simpson RJ, Ji H, Aikawa E, Altevogt P, Askenase P, Bond VC, Borras FE, Breakefield X, Budnik V, Buzas E, Camussi G, Clayton A (2012). Vesiclepedia: a compendium for extracellular vesicles with continuous community annotation. PLoS Biol.

[4] Raposo G, Stoorvogel W (2013). Extracellular vesicles: exosomes, microvesicles,, friends. J Cell Biol.

[5] Mathivanan S, Ji H, Simpson RJ (2010). Exosomes: extracellular organelles important in intercellular communication. Journal of proteomics.

[6] Cocucci E, Racchetti G, Meldolesi J (2009). Shedding microvesicles: artefacts no more. Trends Cell Biol.

[7] Crescitelli R, Lasser C, Szabo TG, Kittel A, Eldh M, Dianzani I, Buzas EI, Lotvall J (2013). Distinct RNA profiles in subpopulations of extracellular vesicles: apoptotic bodies, microvesicles and exosomes. Journal of extracellular vesicles.

[8] Miranda KC, Bond DT, McKee M, Skog J, Paunescu TG, Da Silva N, Brown D, Russo LM (2010). Nucleic acids within urinary exosomes/microvesicles are potential biomarkers for renal disease. Kidney Int.

[9] Nilsson J, Skog J, Nordstrand A, Baranov V, Mincheva-Nilsson L, Breakefield XO, Widmark A (2009). Prostate cancer-derived urine exosomes: a novel approach to biomarkers for prostate cancer. Br J Cancer.

[10] Duijvesz D, Luider T, Bangma CH, Jenster G (2011). Exosomes as biomarker treasure chests for prostate cancer. Eur Urol.

[11] Mitchell PJ, Welton J, Staffurth J, Court J, Mason MD, Tabi Z, Clayton A (2009). Can urinary exosomes act as treatment response markers in prostate cancer?. J Transl Med.

[12] Overbye A, Skotland T, Koehler CJ, Thiede B, Seierstad T, Berge V, Sandvig K, Llorente A (2015). Identification of prostate cancer biomarkers in urinary exosomes. Oncotarget.

[13] Hessvik NP, Sandvig K, Llorente A (2013). Exosomal miRNAs as Biomarkers for Prostate Cancer. Frontiers in genetics.

[14] Priest R, Garzotto M, Kaufman J (2012). Benign prostatic hyperplasia: a brief overview of pathogenesis, diagnosis, and therapy. Techniques in vascular and interventional radiology.

[15] Pienta KJ (2009). Critical appraisal of prostate-specific antigen in prostate cancer screening: 20 years later. Urology.

[16] Ugalde-Olano A, Egia A, Fernandez-Ruiz S, Loizaga-Iriarte A, Zuniga-Garcia P, Garcia S, Royo F, Lacasa-Viscasillas I, Castro E, Cortazar AR, Zabala-Letona A, Martin-Martin N, Arruabarrena-Aristorena A (2015). Methodological aspects of the molecular and histological study of prostate cancer: focus on PTEN. Methods.

[17] Kim DK, Lee J, Kim SR, Choi DS, Yoon YJ, Kim JH, Go G, Nhung D, Hong K, Jang SC, Kim SH, Park KS, Kim OY (2015). EVpedia: a community web portal for extracellular vesicles research. Bioinformatics.

[18] Taylor BS, Schultz N, Hieronymus H, Gopalan A, Xiao Y, Carver BS, Arora VK, Kaushik P, Cerami E, Reva B, Antipin Y, Mitsiades N, Landers T (2010). Integrative genomic profiling of human prostate cancer. Cancer Cell.

[19] Grasso CS, Wu YM, Robinson DR, Cao X, Dhanasekaran SM, Khan AP, Quist MJ, Jing X, Lonigro RJ, Brenner JC, Asangani IA, Ateeq B, Chun SY (2012). The mutational landscape of lethal castration-resistant prostate cancer. Nature.

[20] Jarrard DF, Paul R, van Bokhoven A, Nguyen SH, Bova GS, Wheelock MJ, Johnson KR, Schalken J, Bussemakers M, Isaacs WB (1997). P-Cadherin is a basal cell-specific epithelial marker that is not expressed in prostate cancer. Clin Cancer Res.

[21] Rhodes DR, Yu J, Shanker K, Deshpande N, Varambally R, Ghosh D, Barrette T, Pandey A, Chinnaiyan AM (2004). ONCOMINE: a cancer microarray database and integrated data-mining platform. Neoplasia.

[22] Berglund L, Bjorling E, Oksvold P, Fagerberg L, Asplund A, Szigyarto CA, Persson A, Ottosson J, Wernerus H, Nilsson P, Lundberg E, Sivertsson A, Navani S (2008). A genecentric Human Protein Atlas for expression profiles based on antibodies. Mol Cell Proteomics.

[23] Ponten F, Jirstrom K, Uhlen M (2008). The Human Protein Atlas—a tool for pathology. J Pathol.

[24] Uhlen M, Bjorling E, Agaton C, Szigyarto CA, Amini B, Andersen E, Andersson AC, Angelidou P, Asplund A, Asplund C, Berglund L, Bergstrom K, Brumer H (2005). A human protein atlas for normal and cancer tissues based on antibody proteomics. Mol Cell Proteomics.

[25] Uhlen M, Fagerberg L, Hallstrom BM, Lindskog C, Oksvold P, Mardinoglu A, Sivertsson A, Kampf C, Sjostedt E, Asplund A, Olsson I, Edlund K, Lundberg E (2015). Proteomics. Tissue-based map of the human proteome. Science.

[26] Uhlen M, Oksvold P, Fagerberg L, Lundberg E, Jonasson K, Forsberg M, Zwahlen M, Kampf C, Wester K, Hober S, Wernerus H, Bjorling L, Ponten F (2010). Towards a knowledge-based Human Protein Atlas. Nat Biotechnol.

[27] Paredes J, Figueiredo J, Albergaria A, Oliveira P, Carvalho J, Ribeiro AS, Caldeira J, Costa AM, Simoes-Correia J, Oliveira MJ, Pinheiro H, Pinho SS, Mateus R (2012). Epithelial E- and P-cadherins: role and clinical significance in cancer. Biochim Biophys Acta.

[28] Vieira AF, Ricardo S, Ablett MP, Dionisio MR, Mendes N, Albergaria A, Farnie G, Gerhard R, Cameselle-Teijeiro JF, Seruca R, Schmitt F, Clarke RB, Paredes J (2012). P-cadherin is coexpressed with CD44 and CD49f and mediates stem cell properties in basal-like breast cancer. Stem Cells.

[29] Cerami E, Gao J, Dogrusoz U, Gross BE, Sumer SO, Aksoy BA, Jacobsen A, Byrne CJ, Heuer ML, Larsson E, Antipin Y, Reva B, Goldberg AP (2012). The cBio cancer genomics portal: an open platform for exploring multidimensional cancer genomics data. Cancer Discov.

[30] Gao J, Aksoy BA, Dogrusoz U, Dresdner G, Gross B, Sumer SO, Sun Y, Jacobsen A, Sinha R, Larsson E, Cerami E, Sander C, Schultz N (2013). Integrative analysis of complex cancer genomics and clinical profiles using the cBioPortal. Sci Signal.

[31] Grisanzio C, Signoretti S (2008). p63 in prostate biology, pathology. J Cell Biochem.

[32] Signoretti S, Waltregny D, Dilks J, Isaac B, Lin D, Garraway L, Yang A, Montironi R, McKeon F, Loda M (2000). p63 is a prostate basal cell marker and is required for prostate development. Am J Pathol.

[33] Weinstein MH, Signoretti S, Loda M (2002). Diagnostic utility of immunohistochemical staining for p63, a sensitive marker of prostatic basal cells. Mod Pathol.

[34] Shimomura Y, Wajid M, Shapiro L, Christiano AM (2008). P-cadherin is a p63 target gene with a crucial role in the developing human limb bud and hair follicle. Development.

[35] Gonzales PA, Pisitkun T, Hoffert JD, Tchapyjnikov D, Star RA, Kleta R, Wang NS, Knepper MA (2009). Large-scale proteomics and phosphoproteomics of urinary exosomes. J Am Soc Nephrol.

[36] Hoorn EJ, Pisitkun T, Zietse R, Gross P, Frokiaer J, Wang NS, Gonzales PA, Star RA, Knepper MA (2005). Prospects for urinary proteomics: exosomes as a source of urinary biomarkers. Nephrology.

[37] Perez A, Loizaga A, Arceo R, Lacasa I, Rabade A, Zorroza K, Mosen-Ansorena D, Gonzalez E, Aransay AM, Falcon-Perez JM, Unda-Urzaiz M, Royo F (2014). A Pilot Study on the Potential of RNA-Associated to Urinary Vesicles as a Suitable Non-Invasive Source for Diagnostic Purposes in Bladder Cancer. Cancers.

[38] Duijvesz D, Burnum-Johnson KE, Gritsenko MA, Hoogland AM, Vredenbregt-van den Berg MS, Willemsen R, Luider T, Pasa-Tolic L, Jenster G (2013). Proteomic profiling of exosomes leads to the identification of novel biomarkers for prostate cancer. PLoS One.

[39] Duijvesz D, Versluis CY, van der Fels CA, Vredenbregt-van den Berg MS, Leivo J, Peltola MT, Bangma CH, Pettersson KS, Jenster G (2015). Immuno-based detection of extracellular vesicles in urine as diagnostic marker for prostate cancer. Int J Cancer.

[40] Baek S, Lee YW, Yoon S, Baek SY, Kim BS, Oh SO (2010). CDH3/P-Cadherin regulates migration of HuCCT1 cholangiocarcinoma cells. Anat Cell Biol.

[41] Bauer R, Valletta D, Bauer K, Thasler WE, Hartmann A, Muller M, Reichert TE, Hellerbrand C (2014). Downregulation of P-cadherin expression in hepatocellular carcinoma induces tumorigenicity. Int J Clin Exp Pathol.

[42] Hibi K, Goto T, Mizukami H, Kitamura YH, Sakuraba K, Sakata M, Saito M, Ishibashi K, Kigawa G, Nemoto H, Sanada Y (2009). Demethylation of the CDH3 gene is frequently detected in advanced colorectal cancer. Anticancer Res.

[43] Hibi K, Kitamura YH, Mizukami H, Goto T, Sakuraba K, Sakata M, Saito M, Ishibashi K, Kigawa G, Nemoto H, Sanada Y (2009). Frequent CDH3 demethylation in advanced gastric carcinoma. Anticancer Res.

[44] Lysne D, Johns J, Walker A, Ecker R, Fowler C, Lawson KR (2014). P-cadherin potentiates ligand-dependent EGFR and IGF-1R signaling in dysplastic and malignant oral keratinocytes. Oncol Rep.

[45] Milicic A, Harrison LA, Goodlad RA, Hardy RG, Nicholson AM, Presz M, Sieber O, Santander S, Pringle JH, Mandir N, East P, Obszynska J, Sanders S (2008). Ectopic expression of P-cadherin correlates with promoter hypomethylation early in colorectal carcinogenesis and enhanced intestinal crypt fission *in vivo*. Cancer Res.

[46] Paredes J, Albergaria A, Oliveira JT, Jeronimo C, Milanezi F, Schmitt FC (2005). P-cadherin overexpression is an indicator of clinical outcome in invasive breast carcinomas and is associated with CDH3 promoter hypomethylation. Clin Cancer Res.

[47] Sousa B, Ribeiro AS, Nobre AR, Lopes N, Martins D, Pinheiro C, Vieira AF, Albergaria A, Gerhard R, Schmitt F, Baltazar F, Paredes J (2014). The basal epithelial marker P-cadherin associates with breast cancer cell populations harboring a glycolytic and acid-resistant phenotype. BMC Cancer.

[48] Taniuchi K, Nakagawa H, Hosokawa M, Nakamura T, Eguchi H, Ohigashi H, Ishikawa O, Katagiri T, Nakamura Y (2005). Overexpressed P-cadherin/CDH3 promotes motility of pancreatic cancer cells by interacting with p120ctn and activating rho-family GTPases. Cancer Res.

[49] Yi S, Yang ZL, Miao X, Zou Q, Li J, Liang L, Zeng G, Chen S (2014). N-cadherin and P-cadherin are biomarkers for invasion, metastasis, and poor prognosis of gallbladder carcinomas. Pathol Res Pract.

[50] Mandeville JA, Silva Neto B, Vanni AJ, Smith GL, Rieger-Christ KM, Zeheb R, Loda M, Libertino JA, Summerhayes IC (2008). P-cadherin as a prognostic indicator and a modulator of migratory behaviour in bladder carcinoma cells. BJU Int.

[51] Signoretti S, Pires MM, Lindauer M, Horner JW, Grisanzio C, Dhar S, Majumder P, McKeon F, Kantoff PW, Sellers WR, Loda M (2005). p63 regulates commitment to the prostate cell lineage. Proc Natl Acad Sci U S A.

[52] Costa-Silva B, Aiello NM, Ocean AJ, Singh S, Zhang H, Thakur BK, Becker A, Hoshino A, Mark MT, Molina H, Xiang J, Zhang T, Theilen TM (2015). Pancreatic cancer exosomes initiate pre-metastatic niche formation in the liver. Nat Cell Biol.

[53] Peinado H, Aleckovic M, Lavotshkin S, Matei I, Costa-Silva B, Moreno-Bueno G, Hergueta-Redondo M, Williams C, Garcia-Santos G, Ghajar C, Nitadori-Hoshino A, Hoffman C, Badal K (2012). Melanoma exosomes educate bone marrow progenitor cells toward a pro-metastatic phenotype through MET. Nat Med.

[54] Nardella C, Chen Z, Salmena L, Carracedo A, Alimonti A, Egia A, Carver B, Gerald W, Cordon-Cardo C, Pandolfi PP (2008). Aberrant Rheb-mediated mTORC1 activation and Pten haploinsufficiency are cooperative oncogenic events. Genes Dev.

[55] Carracedo A, Weiss D, Leliaert AK, Bhasin M, de Boer VC, Laurent G, Adams AC, Sundvall M, Song SJ, Ito K, Finley LS, Egia A, Libermann T (2012). A metabolic prosurvival role for PML in breast cancer. The Journal of clinical investigation.

[56] Dragovic RA, Gardiner C, Brooks AS, Tannetta DS, Ferguson DJ, Hole P, Carr B, Redman CW, Harris AL, Dobson PJ, Harrison P, Sargent IL (2011). Sizing and phenotyping of cellular vesicles using Nanoparticle Tracking Analysis. Nanomedicine: nanotechnology, biology, and medicine.

[57] Ruiz de Eguino G, Infante A, Schlangen K, Aransay AM, Fullaondo A, Soriano M, Garcia-Verdugo JM, Martin AG, Rodriguez CI (2012). Sp1 transcription factor interaction with accumulated prelamin a impairs adipose lineage differentiation in human mesenchymal stem cells: essential role of sp1 in the integrity of lipid vesicles. Stem Cells Transl Med.

[58] Du P, Kibbe WA, Lin SM (2008). lumi: a pipeline for processing Illumina microarray. Bioinformatics.

[59] Gentleman RC, Carey VJ, Bates DM, Bolstad B, Dettling M, Dudoit S, Ellis B, Gautier L, Ge Y, Gentry J, Hornik K, Hothorn T, Huber W (2004). Bioconductor: open software development for computational biology and bioinformatics. Genome Biol.

[60] Team RDC (2011). R: A Language and Environment for Statistical Computing.

[61] Baca SC, Prandi D, Lawrence MS, Mosquera JM, Romanel A, Drier Y, Park K, Kitabayashi N, MacDonald TY, Ghandi M, Van Allen E, Kryukov GV, Sboner A (2013). Punctuated evolution of prostate cancer genomes. Cell.

[62] Robinson D, Van Allen EM, Wu YM, Schultz N, Lonigro RJ, Mosquera JM, Montgomery B, Taplin ME, Pritchard CC, Attard G, Beltran H, Abida W, Bradley RK (2015). Integrative clinical genomics of advanced prostate cancer. Cell.

