# Supplementary material for: Transcriptomic profiling of urine extracellular vesicles reveals alterations of CDH3 in prostate cancer
Source: Oncotarget. 2016 Jan 12;7(6):6835–46. doi: 10.18632/oncotarget.6899 (PMC4872752; doi:10.18632/oncotarget.6899)
Supplement: Supplementary file 1 [file oncotarget-07-6835-s001.pdf]

Transcriptomic profiling of urine extracellular vesicles reveals alterations of CDH3 in prostate cancer

Supplementary Materials

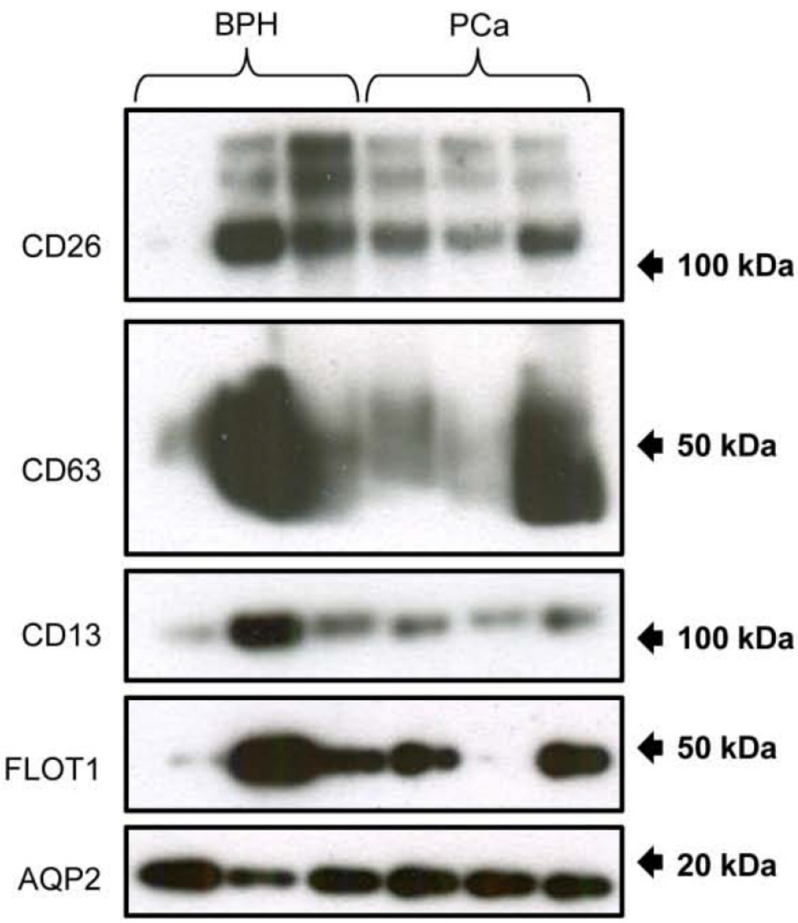

Supplementary Figure S1: Western blot analysis of standard EV markers for the characterization of BPH and PCa uEV extracts (*n* = 3).

A

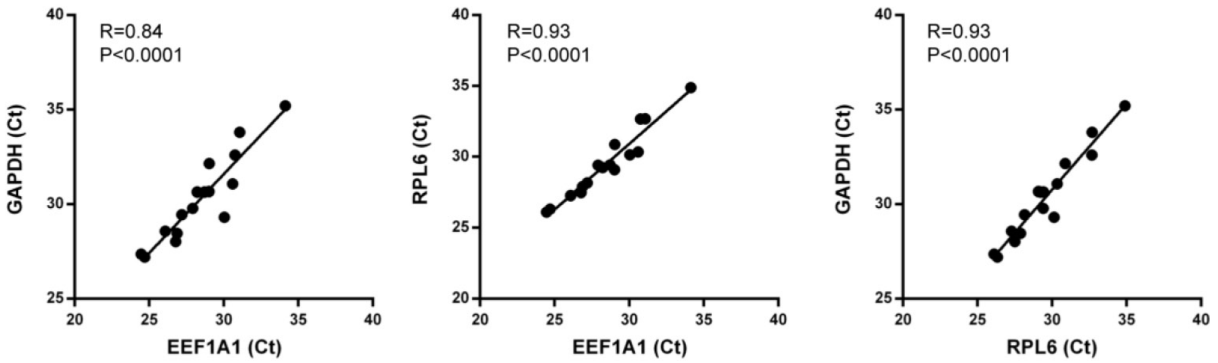

B

RNase treated

	CMTM3	CDH3	EEF1A1	RPL6	GAPDH
**#1**	31,784	36,738	29,163	30,198	30,125
**#2**	35,036	27,578	27,358	26,378	25,462
**#3**	36,475	33,905	30,509	29,315	29,012

C

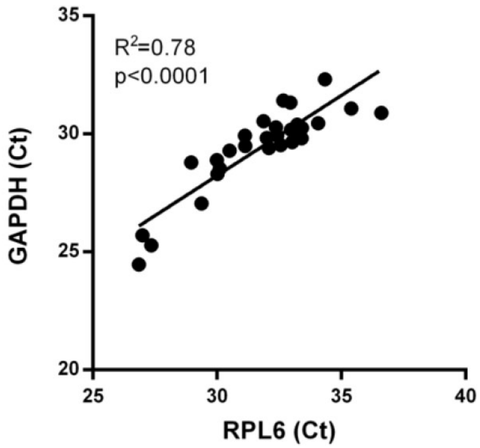

Supplementary Figure S2: (**A**) Correlation analysis of the expression of EEF1A1, RPL6 and GAPDH in uEV samples purified by ultracentrifugation (***n*** = 16). (**B**) mRNA abundance by qRTPCR in RNase treated uEV extracts. Numbers show amplification (Ct) obtained in each qRTPCR reaction. UD: undetermined. (A). (**C**) Correlation analysis of the expression of RPL6 and GAPDH in uEV samples purified with Norgen method (*n* = 28). Statistic test: Pearson´s coefficient (R).

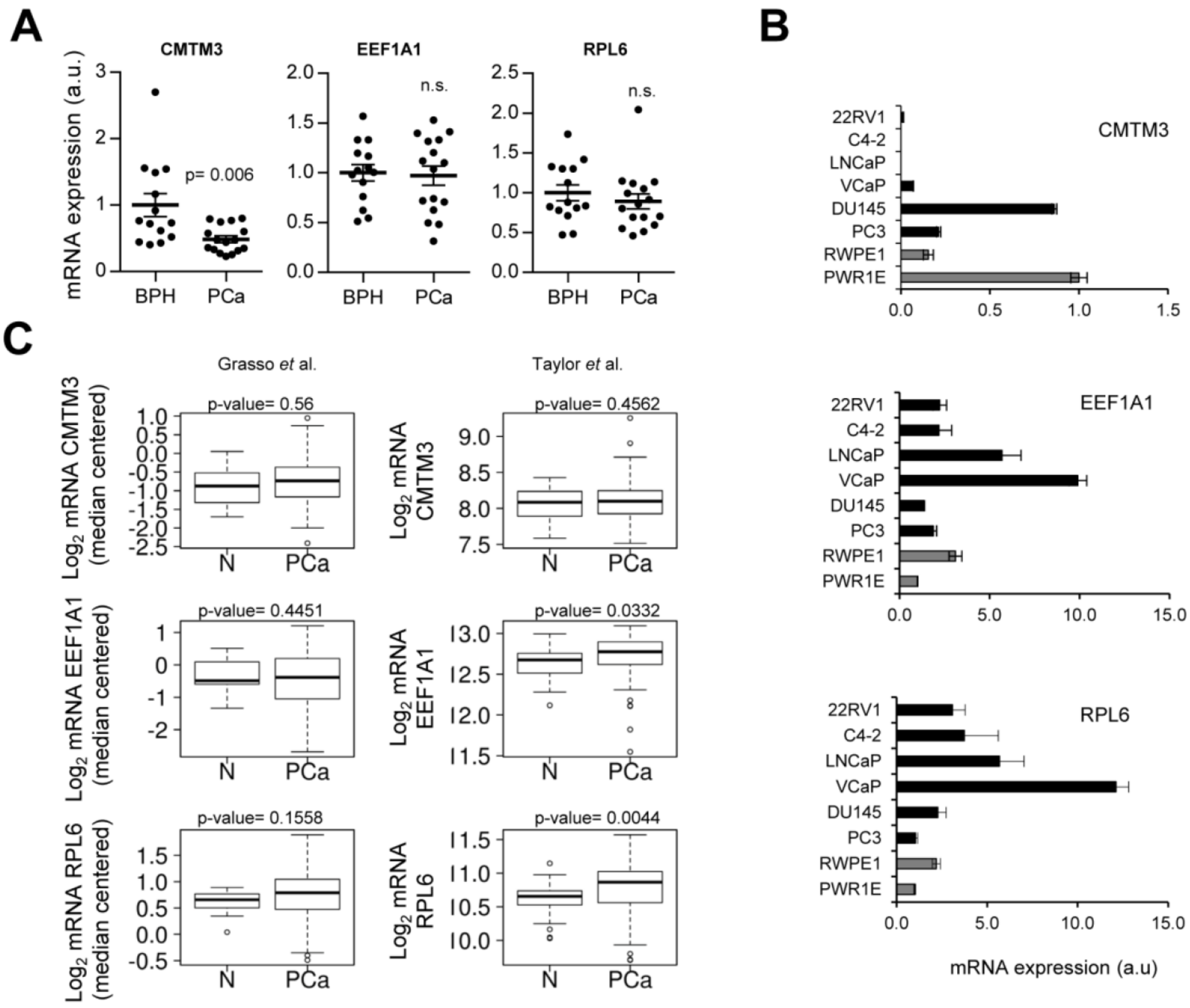

D

Bladder cancer	Comparision	Fold change	p-value
Sanchez-Carbayo et al.	IBUC vs N	13,338	1,39E-15
Lee JS et al.	SBC vs N	1,672	3,32E-06
Dyrskjot et al.	IBUC vsS N	3,014	5,82E-04
	SBC vs N	1,209	6,40E-02
Modlich et al.	IBUC vs N	1,283	1,02E-01
	SBC vs N	1,611	1,90E-02

Renal cancer	Comparision	Fold change	p-value
Yusenko et al.	CRCC vs N	-24,014	0,003
	CCRCC vs N	-16,12	0,005
	RO vs N	-15,496	0,006
	PRCC vs N	-13,881	0,006
Cutcliffe et al.	RWT vs N	-8,673	7,46E-09
	CCCK vs N	-6,468	8,21E-05
Gumz et al.	CCRCC vs N	-3,465	3,25E-05
Jones et al.	PRCC vs N	-2,426	9,53E-09
Beroukhim et al.	hCCRCC vs N	-2,233	4,16E-04
	non-hCCRCC vs N	-2,217	4,28E-04
Lenburg et al.	CCRCC vs N	-1,079	2,07E-01

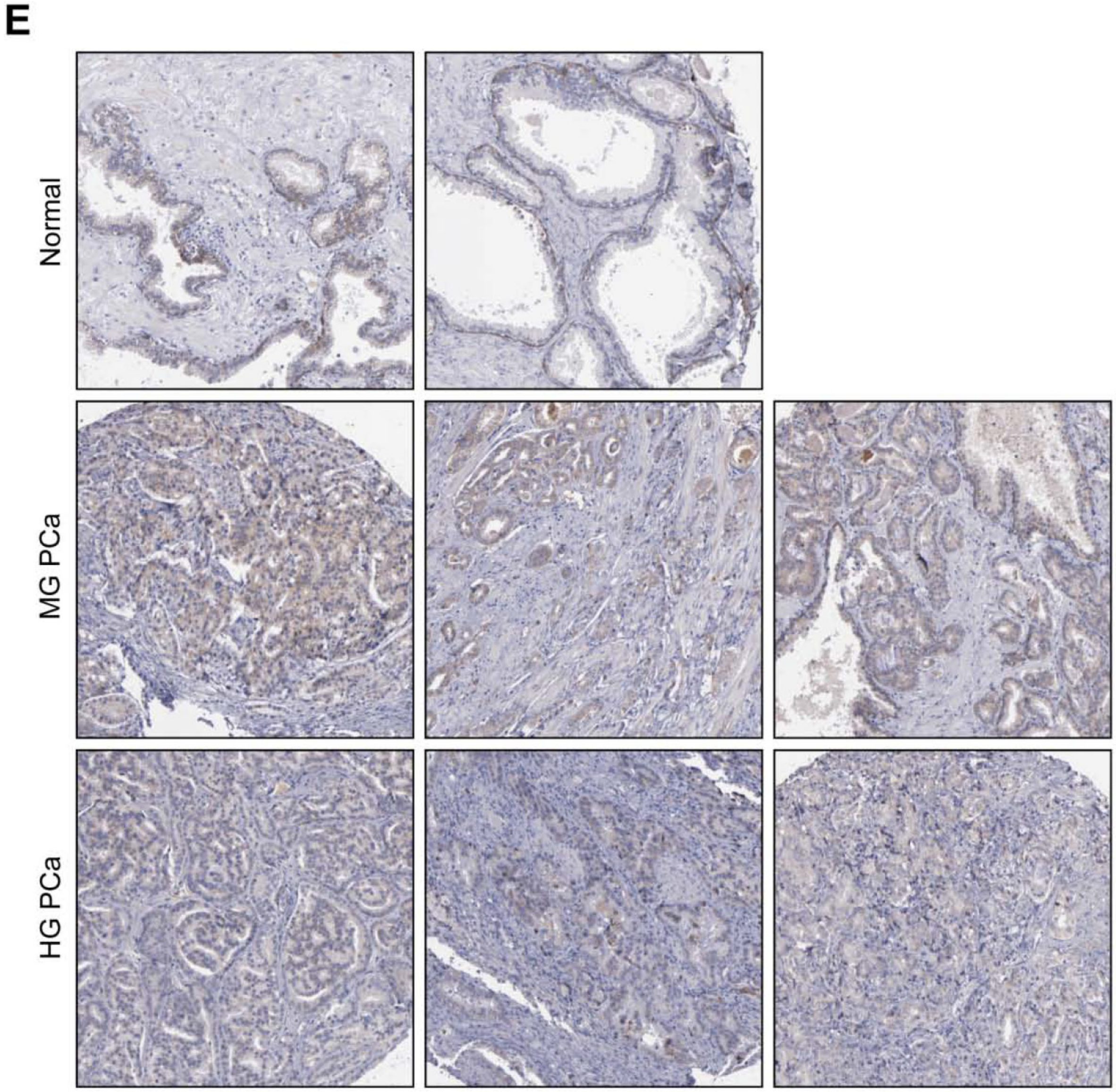

Supplementary Figure S3: (**A**) CMTM3, EEF1A1 and RPL6 expression in tissue biopsies from BPH and PCa. mRNA expression relative to GAPDH is shown. *n* = 14 for BPH and *n* = 16 for PCa. (**B**) CMTM3, EEF1A and RPL6 expression in a panel of metastatic prostate cancer cell lines (black bars) and benign immortalized prostate cell lines (grey bars) relative to beta-Actin. *n* = 3. (**C**) CMTM3, EEF1A and RPL6 expression in two PCa databases (Taylor PCa *n* = 150, normal *n* = 29; Grasso PCa *n* = 76, normal *n* = 12). (**D**) Differential expression analysis of CDH3 in bladder (upper panel) and kidney (lower panel) cancer (data source: Oncomine). (**E**)Additional images of CDH3 staining in normal, medium grade (MG) and high grade (HG) PCa (data source: Human Protein Atlas). IBUC: Inflitrating bladder urothelial carcinoma; SBC: Superficial bladder cancer; CRCC: Chromophobe Renal Cell Carcinoma; CCRCC: Clear Cell Renal Cell Carcinoma; RO: Renal Oncocytoma; PRCC: Papillary Renal Cell Carcinoma; RWT: Renal Wilms tumor; CCCK: Clear cell carcinoma of the kindney; hCCRCC: Hereditary Clear Cell Renal Cell Carcinoma; non-hCCRCC: non-Hereditary Clear Cell Renal Cell Carcinoma. Statistic test: Mann Whitney *U* test (A), Student *t* Test (C and D).

Supplementary Table S1: Patient, tumor and urine sample information

Supplementary Table S2: Universal probe library (Roche) primers and probes employed in this study

Gene Name	Primer Seq Left	Primer Seq Right	Probe #
**CDH3**	tggactttctctctggaatgg	ttctctaacgttttgaagatagcatt	79
**CMTM3**	ctttcctctgctctctcaaagg	tgatgaatgagagacccgact	18
**EEF1A1**	gtgatgctgccattgttgat	agcgacccaaaggtggata	72
**FKBP6**	acggaattagtccttggcttc	tgttgactcagggacattcg	23
**LOC100129121**	ctggtcacacaccgatgc	atggggagcctcctttga	23
**LOC100131541**	ggtggcactcgcctgtag	ccaggctcaggtgattctct	30
**LOC342892**	tgtcatttattattctccatgtttttg	tcctccatcttcaccctctc	38
**LOC654155**	gggccatagttctccctgat	gggagcttcagacacaccat	40
**NACA2**	tctacaagagccctgcttcg	gctgctagttgtgcttgctg	60
**PTER**	ggcacaggggagactaaaaa	cactctagcctgggaagcag	7
**RPL6**	tacggagcagcgcaagat	tccattcgtcagagcaaaca	73
**TES**	tgcctatgcatttactctttcct	ccaacttacaaagagaatactgaaggt	13

Supplementary Table S3: Detection *p*-value in the transcriptomic analysis

Supplementary Table S4: Normalized abundance of transcripts with a fold change between BPH and PCa not greater than 5% and a *T*-test *p*-value above 0.95

Gene Symbol	AVERAGE CANCER	SD CANCER	AVERAGE Hiperplasia	SD Hiperplasia	Fold	*t*-test
AKR1B1	0.000193751	0.000145868	0.000199844	0.000138244	0.969509422	0.965792211
BASP1	0.000412914	0.000297349	0.000405705	0.000163545	1.017768477	0.97100258
C6orf170	0.000192963	1.9187E-05	0.000188934	0.000126496	1.021328905	0.968519107
CDK5RAP3	0.000198292	5.29075E-05	0.000200076	0.000116161	0.99108401	0.981995327
CMIP	0.000328309	0.000129718	0.000328632	4.239E-06	0.999017823	0.997513065
CTSF	0.00147788	0.000871588	0.001522958	0.001515136	0.970400508	0.953297877
EEF1A1	0.003358218	0.002749496	0.003310846	0.00203432	1.014307994	0.977348909
EIF4H	0.000284539	0.00023222	0.000286467	5.14555E-05	0.993271827	0.988953867
GNPTAB	0.000371094	0.000179185	0.000369016	0.000120738	1.005630744	0.986562096
HES4	0.000171792	0.000114082	0.000170317	5.05645E-05	1.00865956	0.987833964
LGMN	0.001044952	0.000503282	0.001025989	0.000481432	1.018483265	0.954147654
LOC100190938	0.00049264	0.000156141	0.000499759	0.000352176	0.985755186	0.966292289
LOC388707	0.000246858	0.000131856	0.000249211	3.95176E-05	0.990559562	0.98242062
LOC643222	0.000208512	8.93909E-05	0.000214968	0.000114263	0.969969466	0.955545743
LOC645387	0.000356233	0.000281467	0.000360863	8.74665E-05	0.987167286	0.984290477
LOC649150	0.001029395	0.001359314	0.001044761	0.000960488	0.985292598	0.987435424
LOC728553	0.0007476	0.000269129	0.000748124	0.000389838	0.999299457	0.998506178
LOC729706	0.000163855	3.68663E-05	0.000162476	0.00010519	1.008489268	0.983615385
LOC731052	0.000197851	0.000136894	0.000196928	0.00014589	1.004687209	0.995386224
MGC71993	0.000252014	4.07632E-06	0.000258149	0.000132193	0.976231819	0.953656711
RPL15	0.000447415	0.000233604	0.000448569	0.000230888	0.997426122	0.996004657

RPL38	0.000544904	0.000408255	0.000534786	3.42236E-05	1.018920058	0.975250582
RPL41	0.001762907	0.001561834	0.001818023	0.00108215	0.969683271	0.952907868
RPL6	0.002643182	0.002352037	0.002727626	0.001600533	0.969041336	0.951900739
RPS16	0.00204265	0.001693695	0.002105452	0.001384853	0.970172068	0.952558808
RPS27	0.001714828	0.001476588	0.001695574	0.001019924	1.011355512	0.98257687
SAT1	0.000293093	0.000100158	0.000289978	8.22434E-05	1.010741239	0.973484044
SRRM1	0.000255995	0.000145271	0.000259192	0.000124457	0.987666083	0.978294597
TXNIP	0.000276496	0.000140165	0.000275402	0.000136109	1.003971702	0.993193138
VN1R2	0.00020661	0.000163864	0.000209499	0.000157302	0.986212378	0.985617061
